# Potential distribution, range dynamics, and livestock exposure risk of *Veratrum nigrum* L. in China under climate change

**DOI:** 10.3389/fpls.2026.1835288

**Published:** 2026-05-13

**Authors:** Shuoning Zhang, Wenjing Xu, Lingfeng Ma, Yiwei Shen, Weitao Yao, Wenzhuo Zhang, Alyaa Nasr, Yang Nan

**Affiliations:** 1 Liaoning University of Traditional Chinese Medicine, Shenyang, China; 2 Menoufia University, Shebin El-Koom, Egypt

**Keywords:** climate change, livestock risk, MaxEnt, multi-objective optimisation, *Veratrum nigrum* L.

## Abstract

Climate change may alter the spatial distribution of toxic plants and increase their spatial overlap with grazing livestock, thereby posing risks to grassland ecosystems and animal health. *Veratrum nigrum* L., a medicinal plant with strong toxicity, is widely distributed in Eurasian temperate regions and frequently causes livestock poisoning. In this study, we predicted the current and future potential distribution of *V. nigrum* in China and quantified the associated grazing risk. We developed a MaxEnt model optimised with the multi-objective evolutionary algorithm NSGA-III to balance predictive accuracy, model complexity, and generalisation. The model performed well (AUC = 0.898, TSS = 0.687), and elevation, precipitation of the wettest month, and mean temperature of the driest quarter emerged as the dominant environmental drivers. The current suitable habitat of *V. nigrum* covered 236.17 × 10^4^ km². Under future climate scenarios, the total suitable area is projected to increase by 18-41%, with highly suitable habitat expanding by up to 185% and the distribution centroid shifting south-westward. Spatial risk assessment based on pixel-wise overlap between habitat suitability and livestock density revealed that cattle face the highest exposure risk (83.54 × 10^4^ km²), followed by sheep (80.98 × 10^4^ km²) and goats (68.04 × 10^4^ km²). Risk hotspots were mainly concentrated in central China and the Sichuan Basin. These findings provide a spatially explicit basis for evaluating toxic-plant risk and informing adaptive grazing management under climate change.

## 1 Introduction

Shifts in species distributions not only influence community structure and ecosystem functions but also directly impact biodiversity conservation and ecosystem management. In the context of climate change, predicting future suitable habitats for key species is important for the development of conservation strategies and adaptive management measures (Xu et al., 2025b). As a component of Eurasian temperate ecosystems, changes in the distribution of *Veratrum nigrum* L. are relevant not only to the conservation and utilisation of its germplasm resources but also to grassland ecosystem stability because of its toxic and pharmacologically active constituents.

*Veratrum nigrum* L. (*V. nigrum*), also known as mountain onion or seven-mile-red, is a perennial herbaceous plant belonging to the genus Veratrum within the family Liliaceae. This species is widely distributed across the temperate regions of Eurasia, typically growing at elevations of 1200–3300 metres beneath forests or within grasslands on mountain slopes. The plant can reach a height of 1 metre, featuring a robust rhizome and broad leaves. Its panicle inflorescence bears clusters of purple and black flowers (Zhao et al., 2024b). As a significant toxic medicinal plant within China’s traditional herbal medicine system, Veratrum contains over 137 chemical constituents, including steroidal alkaloids, iridoids, flavonoids, and dipeptides (Liu et al., 2023). Among these, steroidal alkaloids (such as verazine, veratrumine, cyclobamamine and their derivatives) constitute the primary source of its pharmacological activity (Yuan et al., 2023). Modern pharmacological research indicates that these alkaloids hold potential applications in hypotensive, anti-inflammatory, anti-platelet aggregation, anti-adipogenic, and insecticidal effects (Cai et al., 2019; Zheng et al., 2019; Zhou et al., 2023). However, such bioactive compounds often carry inherent toxicity. Accidental ingestion by livestock frequently leads to acute poisoning, miscarriage, or even mortality, posing a tangible threat to production safety in animal husbandry (Aboling, 2023). Thus, *V. nigrum* possesses both medicinal value and toxicity: on the one hand, its active constituents harbour significant therapeutic potential; on the other hand, its inherent toxicity presents a non-negligible exposure risk to grazing animals within grassland ecosystems.

Under ongoing climate change, assessing possible shifts in the suitable habitat of *V. nigrum* is important not only for the conservation and sustainable utilisation of medicinal plant resources but also for reducing the exposure risk of grazing livestock. Species distribution models (SDMs) provide an effective framework for predicting current and potential species ranges by quantifying relationships between species occurrence records and environmental variables, and they have been widely applied in biogeography, conservation ecology, invasive species assessment, and studies of climate-change-driven distribution shifts (Elith and Leathwick, 2009; Özkan Tümer et al., 2026). Common SDM approaches include traditional statistical models such as generalised linear models (GLMs) and generalised additive models (GAMs), as well as machine-learning methods such as random forests (RF) and maximum entropy modelling (MaxEnt). However, these models differ in their assumptions, data requirements, and predictive performance. In particular, some methods depend on reliable absence data, whereas others may be less stable or less suitable when only limited occurrence records are available (Valavi et al., 2022). For example, GBM and RF models are prone to overfitting the training data, whereas GLM models may overpredict into unsampled areas (Ahmadi et al., 2023). In contrast, MaxEnt can effectively use presence-only data and has shown relatively robust predictive performance under small-sample conditions (Elith et al., 2010). Maximum Entropy (MaxEnt) is a species distribution modelling algorithm founded on the principle of maximum entropy, which estimates the least biased probability distribution constrained by known environmental conditions (Phillips et al., 2006). Mathematically, it can be formulated as a log-linear model with L1 (Lasso) regularisation, enabling the estimation of species distribution probabilities within environmental space by maximising the likelihood of occurrence records (Phillips and Dudík, 2008). Owing to these advantages, MaxEnt was selected in this study to predict the potential distribution of *V. nigrum*.

Recent MaxEnt studies have commonly used three R packages for hyperparameter optimisation: SDMtune, ENMeval, and Kuenm. By refining FC (Feature Class) and RM (Regularisation Multiplier), these packages significantly enhance prediction accuracy (Xu et al., 2025a). However, current mainstream optimisation methods still exhibit two limitations: (1) Inefficiency: Traditional grid search approaches (kuenm and ENMeval) employ exhaustive strategies (Cobos et al., 2019; Kass et al., 2021), resulting in poor computational efficiency. (2) Monolithic optimisation objectives: The genetic algorithms (GA) in SDMtune primarily use AUC (Area Under the ROC Curve) as the sole optimisation metric (Vignali et al., 2020), neglecting model complexity (AICc, corrected Akaike Information Criterion) and overfitting-related metrics such as test OR_10_ (10% omission rate) and TSS (True Skill Statistic). Using AUC alone as an evaluation metric for niche models may lead to variable redundancy and diminished ecological interpretability (Xu et al., 2025b). Multi-objective optimisation (MOO) offers a practical way to address these limitations (Ehrgott et al., 2026). NSGA-III (Non-dominated Sorting Genetic Algorithm III), as an evolutionary algorithm tailored for high-dimensional multi-objective problems, can identify Pareto optimal solutions among multiple objectives. It has been extensively applied in engineering and computational optimisation domains (La et al., 2025; Li et al., 2025). Its integration into MaxEnt parameter optimisation enables simultaneous balancing of multiple metrics, achieving a systematic equilibrium between prediction accuracy, model robustness, and computational efficiency, thereby improving model generalisation and computational efficiency.

This study had three objectives. First, we developed an NSGA-III–MaxEnt multi-objective optimisation framework to balance model performance and computational efficiency. Second, we identified the dominant climatic variables influencing the geographical distribution of *V. nigrum* and examined their threshold characteristics. Third, we simulated changes in suitable habitat for *V. nigrum* under different climate scenarios (SSPs, Shared Socioeconomic Pathways) and assessed the potential threat to grassland ecosystems. Methodologically, this research extends the application of multi-objective evolutionary optimisation (MOO) within niche modelling and provides a scientific basis and decision support for managing *V. nigrum* under global change.

## 2 Materials and methods

**Figure 1:**
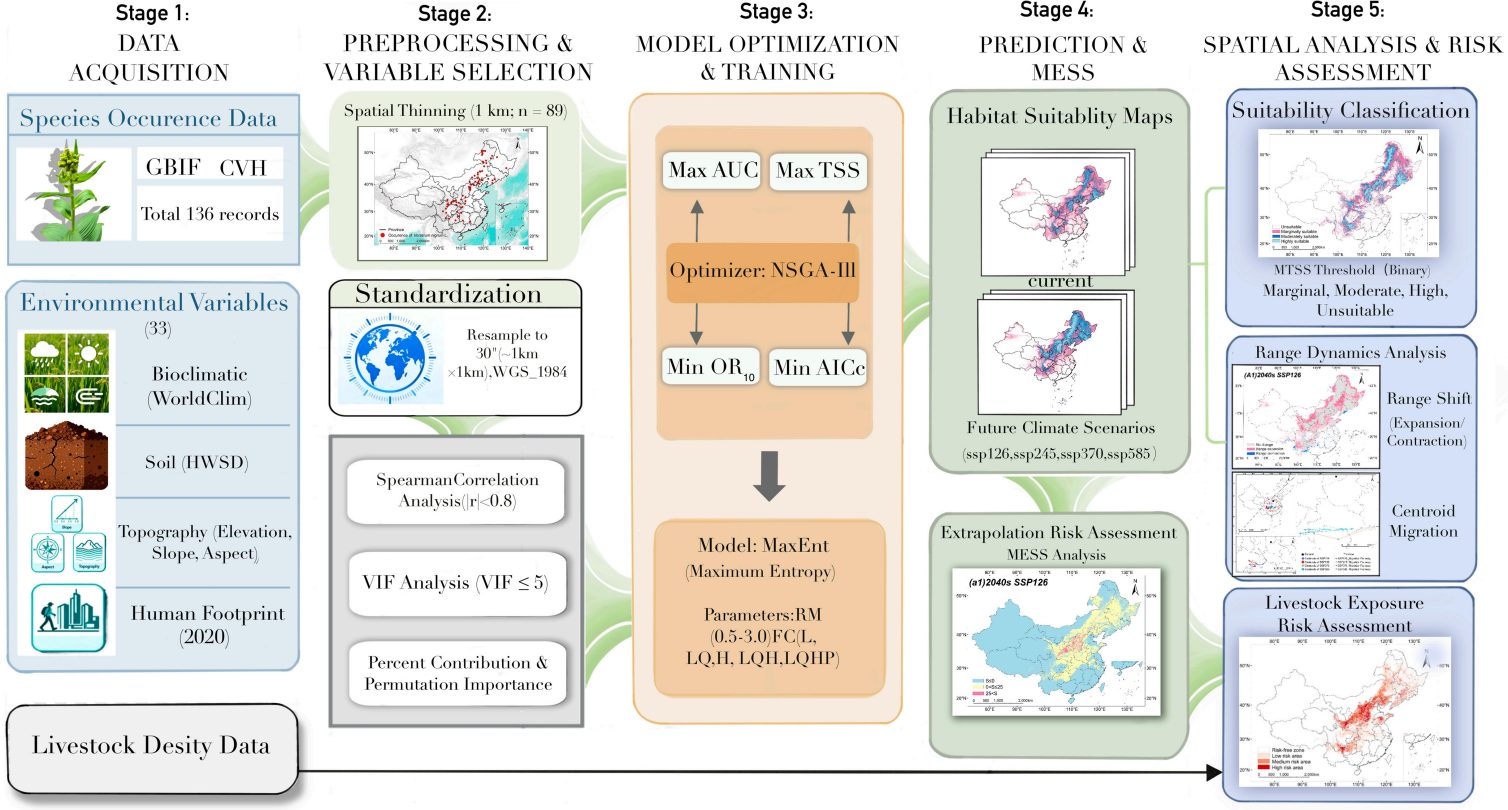
The framework of this study’s contribution.

To better illustrate our research approach, **Figure 1** presents a comprehensive and detailed framework.

### 2.1 Species distribution points

**Figure 2:**
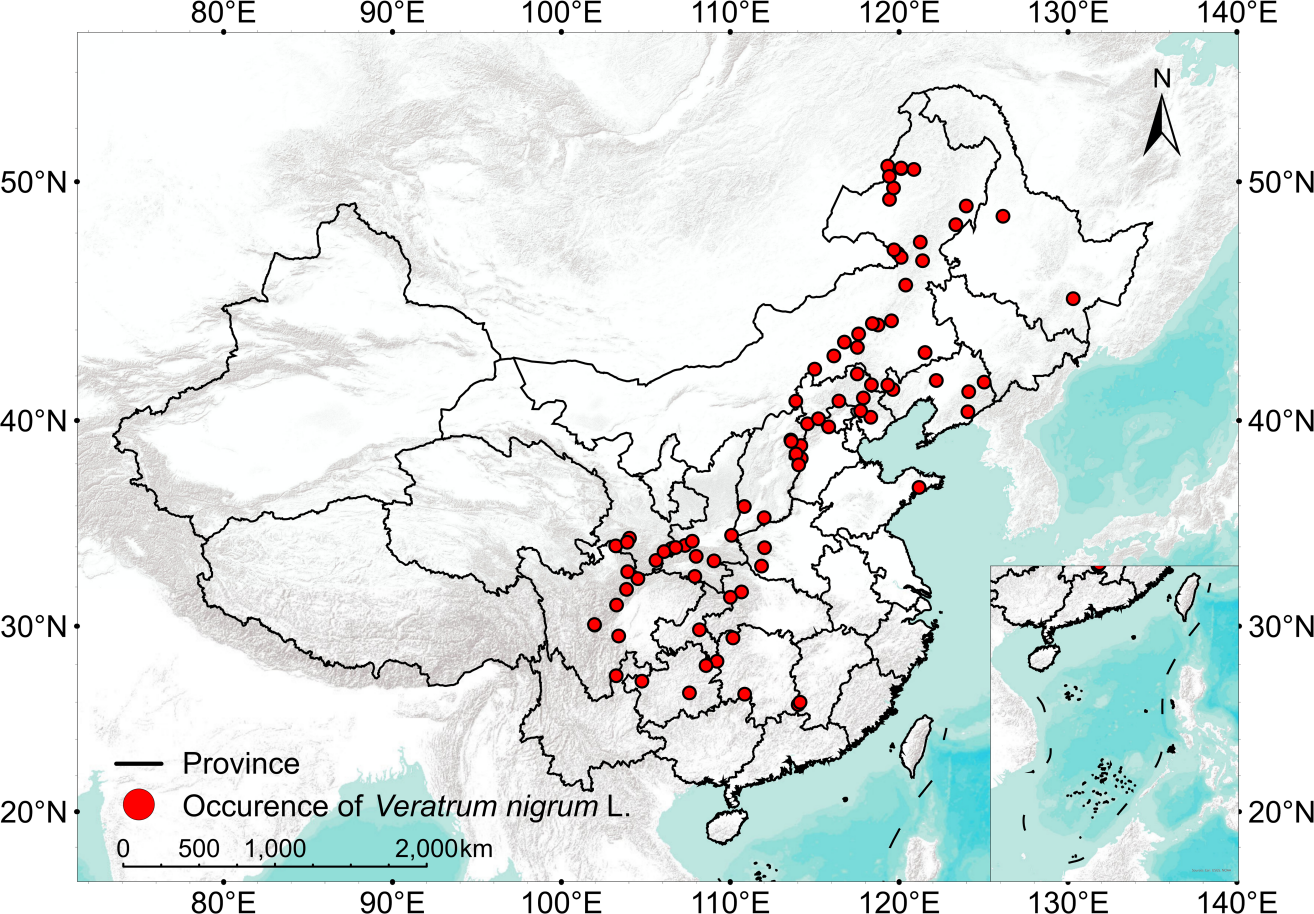
Records of occurrence of *V. nigrum* in the study area.

This study utilised 136 occurrence records collected from the Global Biodiversity Information Facility (GBIF, https://www.gbif.org/) and the Chinese Virtual Herbarium (CVH, https://www.cvh.ac.cn/). To reduce spatial autocorrelation among distribution points, spatial thinning was performed using the R package spThin (Aiello‐Lammens et al., 2015), ensuring a minimum spatial separation of 1 km. This yielded 89 spatially independent occurrence points (**Figure 2**) which were used for subsequent MaxEnt modelling and multi-objective parameter optimisation.

### 2.2 Environmental variables

A total of 33 environmental variables were selected for this study, covering four categories: bioclimatic conditions, soil properties, topography, and anthropogenic influences (**Supplementary Table 1**). The bioclimatic dataset consisted of 19 variables obtained from WorldClim v2.1 (Fick and Hijmans, 2017). Future climate data were derived from the Coupled Model Intercomparison Project Phase 6 (CMIP6) (Eyring et al., 2016), using the Beijing Climate Centre Climate System Model (BCC-CSM2-MR) (Wu et al., 2019). Four Shared Socioeconomic Pathways (SSPs): SSP126, SSP245, SSP370, and SSP585, covering four periods from 2021 to 2100 (Riahi et al., 2017). Ten soil variables were sourced from the Harmonised World Soil Database (HWSD v2.0). Three topographic variables (elevation, slope, aspect) were sourced from the China Resource and Environment Science Data Platform (https://www.resdc.cn/), with slope and aspect derived using ArcGIS processing; the Human Footprint Index (2020) was employed to quantify human activity intensity (Mu et al., 2022). To ensure data consistency, all variables were uniformly resampled to a 30 arc-second resolution (approximately 1 km × 1 km), with the geographic coordinate system standardised to WGS_1984 (Gao et al., 2026).To enhance model generalisation and prevent overfitting, this study employs a multi-method integrated evaluation strategy. Initially, 33 candidate environmental variables were input into the MaxEnt model for preliminary modelling to obtain each variable’s percent contribution and permutation importance. Simultaneously, Spearman correlation analysis was conducted using the R package Hmisc to calculate variable correlations (Harrell, 2026). When |r| ≥ 0.8 and the correlation was statistically significant (P < 0.05), the variables were deemed strongly correlated. Furthermore, the usdm package was employed to calculate the variance inflation factor (VIF) (Babak Naimi, 2023), identifying multicollinearity among candidate environmental variables. A VIF > 5 was deemed indicative of multicollinearity. Based on the combined results of Spearman correlation analysis, VIF diagnostics, and MaxEnt variable importance, variables with strong explanatory power and low collinearity were retained for model construction.

### 2.3 Principles, optimisation and evaluation of the MaxEnt model

Model complexity is mainly controlled by the regularisation multiplier (RM) and feature classes (FC). The RM suppresses model overfitting, while the FC determines the functional form of the relationship between environmental variables and species response (Merow et al., 2013). Given the high sensitivity of model performance to parameter settings (Warren and Seifert, 2011), this study formulated the parameter tuning process as a quadruple-objective optimisation problem and employed the third-generation non-dominated sorting genetic algorithm (NSGA-III) for automatic optimisation. NSGA-III maintains population diversity in high-dimensional objective spaces through a reference-point-based selection mechanism. This makes it more suitable for multi-objective optimisation scenarios than NSGA-II, which relies on the crowding-distance approach (Deb and Jain, 2014). The four optimisation objectives defined in this study were: maximising AUC to reflect overall discrimination between presence and background points; maximising TSS to evaluate sensitivity and specificity beyond the limitations of AUC under varying prevalence; minimising the 10% test omission rate (OR_10_) to reduce overfitting and improve generalisation; and minimising AICc to balance model fit and complexity. The search space was defined with RM ranging from 0.5 to 3.0 (step size 0.1), while FC encompassed five combinations: L, LQ, H, LQH, and LQHP (Zhao et al., 2024a). Each parameter set was run 10 times. Using the bootstrap resampling protocol, 75% of the samples were randomly selected as the training set, with the remaining 25% used for testing. The maximum iteration count was set to 1000, and the background point count was 10,000. The Jackknife test was employed to evaluate the contribution and independent explanatory power of each environmental variable (Wu et al., 2025), thereby enhancing the model’s interpretability. Ultimately, a uniformly distributed Pareto-optimal front was obtained via NSGA-III to achieve a balance between prediction accuracy, stability, and model complexity.

To assess the extrapolation reliability of model predictions under future climate scenarios, this study employs Multivariate Environmental Similarity Surface (MESS) analysis. This method identifies potential extrapolation regions by comparing differences between future environmental conditions and the current training data’s environmental range. When the MESS value S > 0, it indicates that all environmental variables within that region fall within the range of values observed in the training data. In such cases, the model prediction constitutes interpolation, and the results are relatively reliable. Conversely, when S ≤ 0, it signifies that at least one environmental variable exceeds the range of values observed in the training data. Here, the model prediction constitutes extrapolation, and its reliability is diminished. A smaller S value indicates greater deviation of environmental conditions from the training data range and higher prediction uncertainty. Therefore, this study combines MESS analysis results to identify potential environmental extrapolation zones, providing a crucial reference for interpreting future distribution predictions.

### 2.4 Classification and dynamic changes in potential habitat suitability under different climate scenarios

To convert MaxEnt continuous probability predictions into interpretable suitability grades, this study employs the Maximum Training Sensitivity plus Specificity (MTSS) threshold. This method categorises predictions into suitable and unsuitable zones. This thresholding method effectively balances the risks of underprediction and overprediction by maximising the sum of sensitivity and specificity. It is widely applied in species distribution modelling threshold selection (Liu et al., 2013). Building upon this, the suitability range above the MTSS threshold was used to stratify suitable habitat. The “Reclassify” tool in ArcGIS Pro was utilised to process the raster data according to this stratification. The potential distribution of *V. nigrum* was ultimately categorised into four tiers: Highly suitable habitat (P ≥ 0.684789), Moderately suitable habitat (0.523362 ≤ P < 0.684789), Marginally suitable habitat (0.312941 ≤ P < 0.523362), and Unsuitable habitat (P < 0.312941).

To analyse the dynamic shifts in the suitable habitat of *V. nigrum* under future climate scenarios, this study employed spatial overlay analysis using the SDMToolbox v2.0 (Brown, 2014). This compared suitable and unsuitable habitats across different time periods, categorising change types into three classes: no change, range contraction, and range expansion. Concurrently, the geographical centroids of suitable habitats across periods were calculated to spatially quantify the migration direction and distance of *V. nigrum*’s potential distribution centre, revealing its spatial response trends to climate change (Li et al., 2026).

### 2.5 Integration of species distribution and livestock density for risk assessment

The original distribution density data for cattle, sheep, and goats (1 km resolution) were already at the same spatial resolution as the predicted *V. nigrum* suitability grids, and thus no additional resampling or processing was required prior to the risk quantification analyses (Parente et al., 2025). To more accurately assess the potential risks of *V. nigrum* expansion to livestock farming in China, environmental extrapolation constraints were applied prior to risk quantification. Specifically, the current distribution of *V. nigrum* suitability was spatially overlaid with Multivariate Environmental Similarity Surface (MESS) analysis results calculated from contemporary climate variables. Grid cells with MESS ≤ 0, indicating environmental conditions outside the training range and thus extrapolation risk, were conservatively assigned a suitability probability of zero.

The MESS-constrained suitability grids were then multiplied pixel-by-pixel with the density maps of cattle, sheep, and goats to quantify the spatial coupling between *V. nigrum* distribution and livestock density (Bi et al., 2025). Risk and Risk-free areas were first delineated: cells with zero suitability probability or zero livestock density were defined as Risk-free areas, while all other cells were classified as risk areas. The Jenks Natural Breaks method, which maximises between-class variance while minimizing within-class variance, is commonly used for risk classification (Rao et al., 2024; Du et al., 2026). It was applied separately for each livestock species to categorise risk areas into three levels: Low risk Area, Medium risk Area, and High risk Area. By performing Jenks classification independently for cattle, sheep, and goats, the resulting risk categories more accurately reflect the spatial distribution characteristics of each species, providing a robust basis for targeted grazing management, early-warning systems, and risk mitigation strategies.

## 3 Results

### 3.1 Model optimisation, performance evaluation, and current potential distribution of *V. nigrum*

Employing the NSGA-III multi-objective optimisation algorithm, this study obtained a uniformly distributed set of Pareto optimal solutions across four objective spaces: AUC, TSS, OR_10_, and AICc. This achieved an effective balance between the model’s discriminative power, generalisation capability, and computational complexity. Within the Pareto optimal solution set, the optimal hyperparameter combination was determined by comprehensively considering prediction accuracy and model simplicity. This combination comprises the feature combination LQ (linear + quadratic) and a regularisation multiplier of RM = 0.6.

In terms of model performance, the training set AUC was 0.898 and the test set AUC was 0.881, indicating the model possesses strong discrimination capability between presence points and background points (an AUC > 0.85 is generally considered to demonstrate good discriminative performance). The negligible overfitting between training and test set AUC values (approximately 0.017) suggests low overfitting and good generalisation ability. The training set TSS was 0.687 and the testing set TSS was 0.653, both exceeding 0.6. This demonstrates that the model achieves a favourable balance between sensitivity and specificity, yielding high predictive accuracy. The minimal discrepancy between training and test set TSS further underscores the model’s stable and reliable performance. The training set OR_10_ equalled 0.10 by definition and thus does not reflect model performance. The test OR_10_ (0.132) was slightly higher than the expected threshold, indicating a modest increase in omission error on independent data. However, the omission rate remained low overall, supporting acceptable generalisation performance.

Overall, the MaxEnt model employing LQ feature combinations and RM = 0.6 demonstrated favourable performance across AUC, TSS, and OR_10_ metrics. This confirms that the NSGA-III multi-objective optimisation framework effectively balances model accuracy and complexity, providing a robust and reliable foundation for subsequent spatial distribution forecasting and scenario analysis.

**Figure 3:**
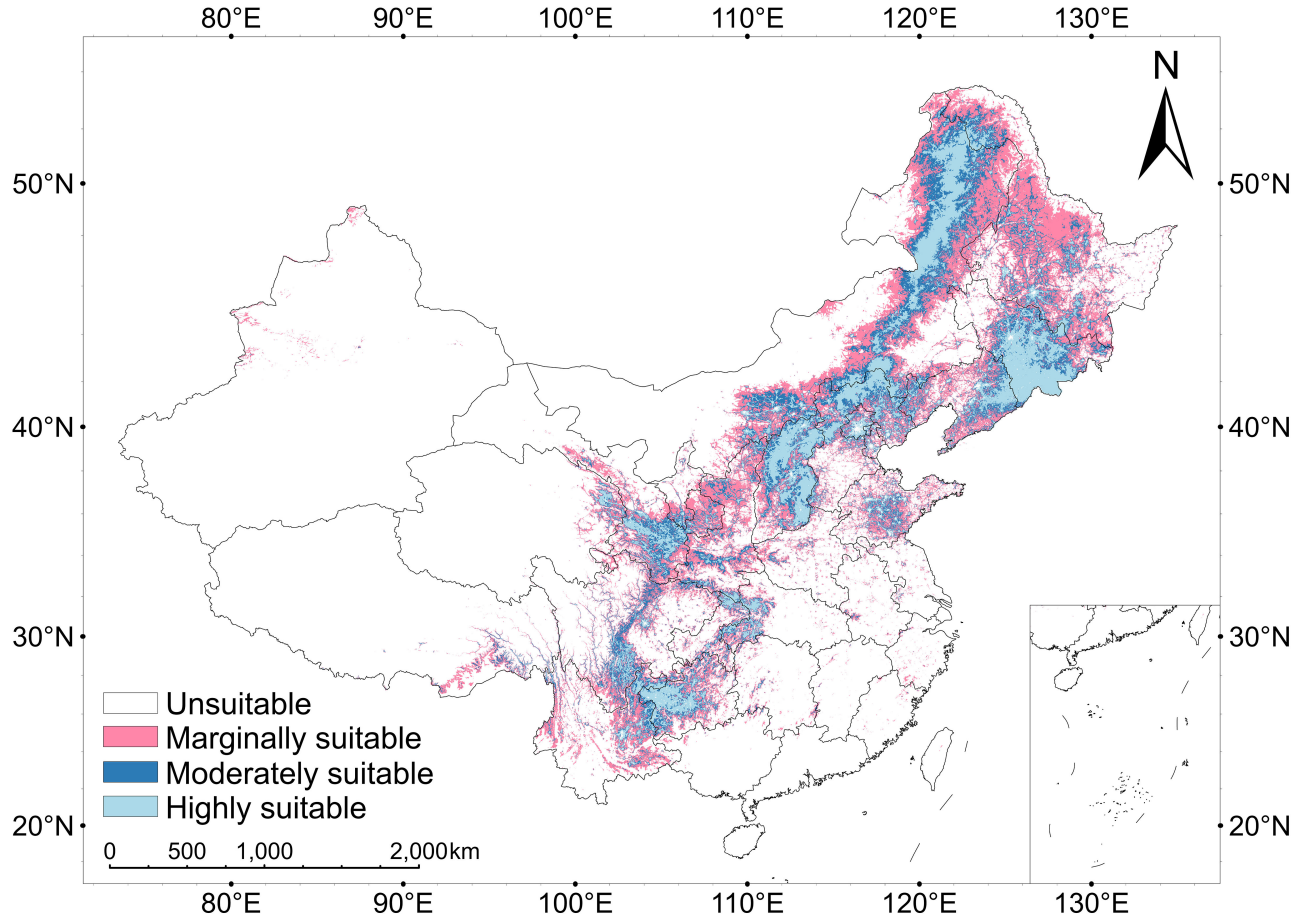
Predicted potential geographic distributions of *V. nigrum* in China.

The study area is categorised into four zones: unsuitable, marginally suitable, moderately suitable, and highly suitable. Results indicate (**Figure 3**) that the current total suitable habitat area for *V. nigrum* is 236.17 × 10^4^ km². Geographically, the Inner Mongolia Autonomous Region and Heilongjiang Province exhibited the largest suitable areas, at 54.32 × 10^4^ km² and 31.48 × 10^4^ km² respectively. Together, these two regions account for 36.32% of the total suitable habitat area, indicating the species’ high ecological suitability within China’s northern grasslands and cold-temperate zones.

Among the different suitability categories, marginally suitable habitat dominates at 118.08 × 10^4^ km²; moderately suitable areas span 59.04 × 10^4^ km², while the highly suitable zone covered 59.04 × 10^4^ km². Marginal suitability accounted for the largest proportion, approximately half of the total suitable area, with moderate and highly suitable zones being comparable in size.

### 3.2 Key environmental drivers

Spearman correlation analysis showed that the pairwise correlations among variables were weak (|r| < 0.8) and statistically significant (P < 0.05) (**Supplementary Figure 1**). The VIF for each variable was below 5, indicating no significant multicollinearity among variables and their suitability for subsequent modelling analysis (**Supplementary Table 2**).

Elevation emerged as the primary variable influencing *V. nigrum* distribution, exhibiting the highest percent contribution and permutation importance (**Table 1**), indicating the species’ sensitivity to altitude. Among climatic variables, bio13 (precipitation of wettest month) and bio9 (mean temperature of driest season) demonstrated high permutation importance, particularly bio13, suggesting that water conditions play a strong independent role in explaining spatial distribution. In contrast, although silt exhibited relatively high contribution during model training, it showed the lowest permutation importance, suggesting limited independent explanatory power and potential information overlap with other variables. The Human Footprint Index (HFP) contributed to the model but demonstrated weaker overall influence compared with climatic and topographic variables.

**Table 1:** Percent contribution and permutation importance of screened environmental variables for *V. nigrum* in China.

Variables	Percent contribution (%)	Permutation importance (%)
Mean Temperature of Driest Quarter (bio9)	10.3	17.2
Precipitation of Wettest Month (bio13)	13.9	36.2
Silt	18.6	1.8
Elevation	41.7	38.3
Human footprint (hfp)	15.5	6.5

**Figure 4:**
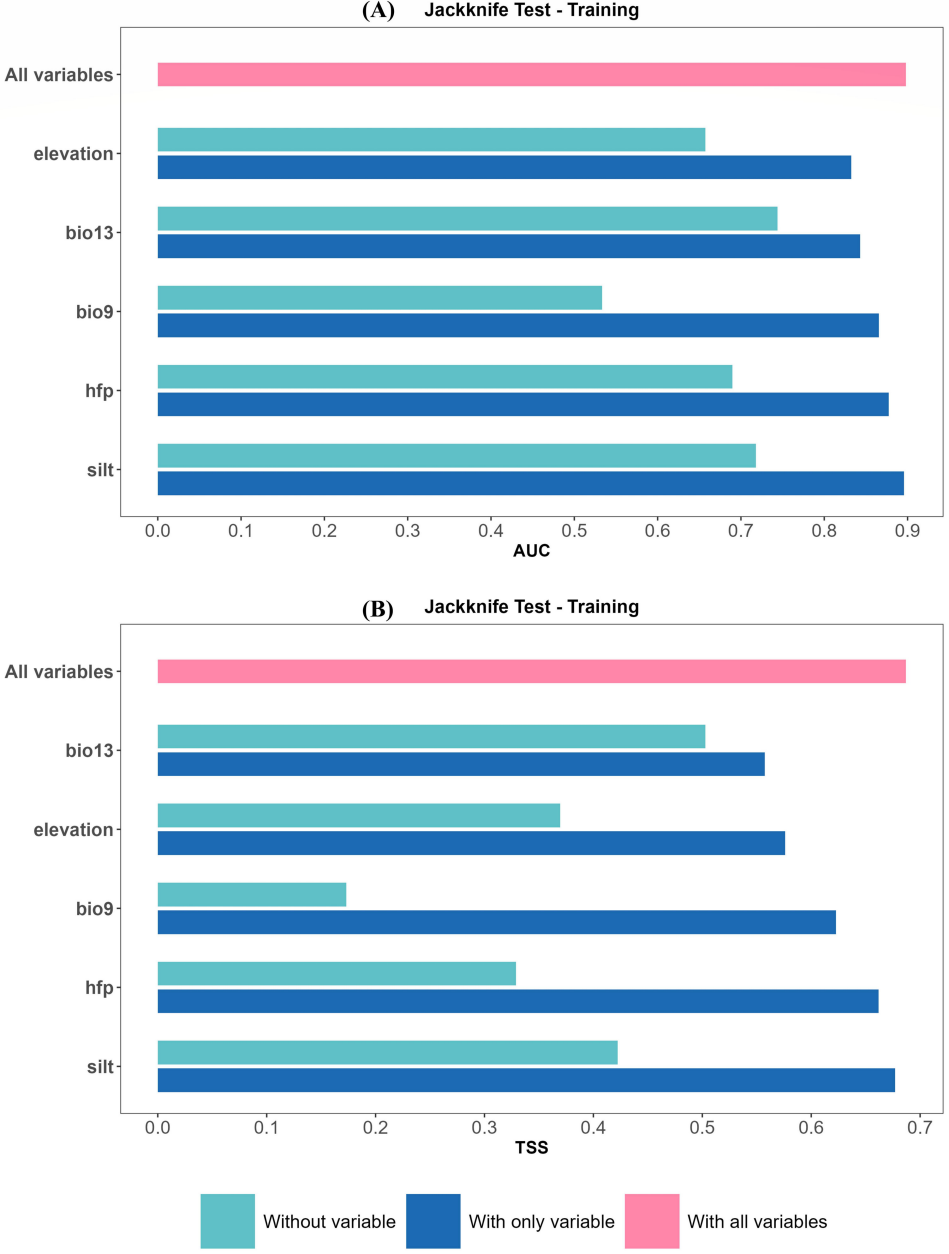
Jackknife test results on the MaxEnt model training dataset with AUC and TSS metrics: **(A)** AUC, **(B)** TSS.

The Jackknife test further indicated (**Figure 4**) that model metrics decline most markedly upon removing bio9, demonstrating its strong independence among multiple environmental variables. Whilst silt performs well in univariate models, its removal in the MaxEnt jackknife “without silt” test yields only a modest decrease in model performance. In summary, the distribution of *V. nigrum* is primarily influenced by the combined effects of climate and topography, with soil variables playing a more supplementary role.

**Figure 5:**
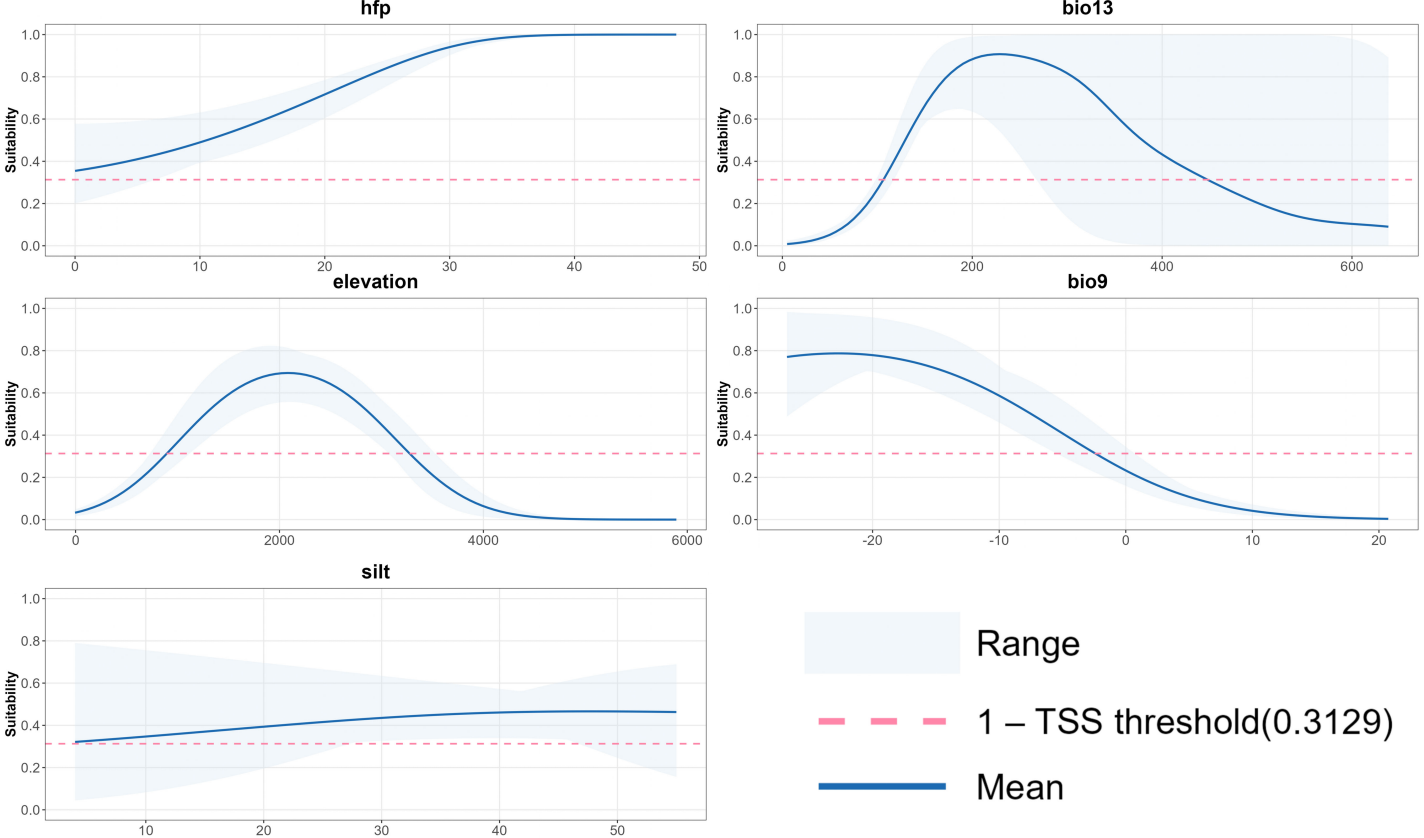
Response curves of the key environmental variables: **(A)** human footprint, **(B)** precipitation of wettest month, **(C)** elevation, **(D)** mean temperature of driest quarter, and **(E)** silt.

Response curve analysis (**Figure 5**) reveals that both bio13 and elevation exhibit distinct peaked response relationships, indicating peak suitability within specific precipitation and elevation ranges. bio9 exhibits a higher probability of suitability at low temperatures, subsequently declining as temperatures rise, reflecting its adaptation to cold, dry-season environments; HFP shows an initial increase followed by a plateauing trend as disturbance intensifies, with overall probabilities consistently above the threshold, indicating a moderate degree of tolerance to anthropogenic disturbance; the response curve for silt is relatively flat, with little variation in suitability probability across different values, further confirming that soil variables play a more secondary, non-dominant influence.

In summary, the spatial distribution of *V. nigrum* is primarily determined by topography and hydrothermal conditions, exhibiting a distinct preference for mid-to-high elevations and cold, humid environments, while soil and human activity variables exert relatively minor influence on its distribution.

### 3.3 Future distribution changes under SSP scenarios

**Figure 6:**
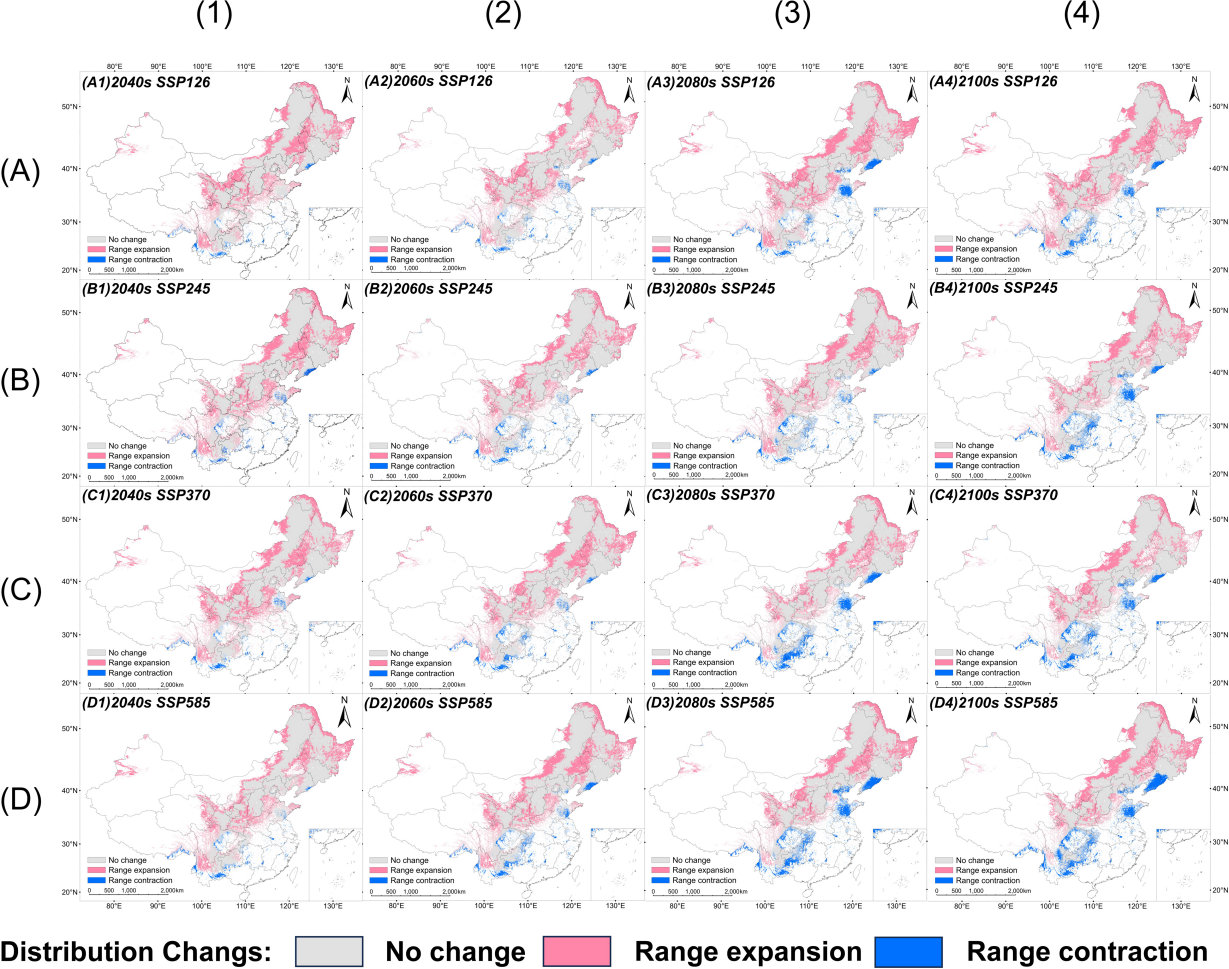
Distribution dynamics of potentially suitable habitats for *V. nigrum* under future climate scenarios: **(A)** SSP126, **(B)** SSP245, **(C)** SSP370, and **(D)** SSP585 climate scenarios; (1) 2040s, (2) 2060s, (3) 2080s, and (4) 2100s.

Compared with the current distribution, the suitable habitat area for *V. nigrum* shows varying degrees of expansion under different climate scenarios at different future time periods (**Figure 6**; **Table 2**). Overall, under SSP126, SSP245, SSP370, and SSP585 scenarios, total suitable habitat area significantly increases compared with current levels, with growth ranging from 18% to 41%, most notably during the 2021–2060 period.

**Table 2:** Changes in suitable habitat distribution of *V. nigrum* under different SSP scenarios.

Scenario	Period		Highly suitable (×10^4^ km²)	Change (%)	Moderately suitable (×10^4^ km²)	Change (%)	Marginally suitable (×10^4^ km²)	Change (%)	Suitability habitat (×10^4^km²)	Change (%)	
	Current	59.04			59.04		118.08		236.17	
SSP126	2040s	168.49		185.37	68.74	16.42	95.45	-19.17	332.68	40.87
	2060s	152.20		157.78	65.65	11.19	96.49	-18.29	314.33	33.10
	2080s	157.57		166.87	70.00	18.56	99.11	-16.07	326.68	38.32
	2100s	161.51		173.56	65.82	11.47	96.56	-18.22	323.89	37.14
SSP245	2040s	159.39		169.97	70.30	19.07	98.00	-17.01	327.70	38.76
	2060s	136.54		131.25	68.76	16.47	103.13	-12.66	308.43	30.60
	2080s	128.12		116.99	71.19	20.57	105.25	-10.87	304.55	28.96
	2100s	129.40		119.16	63.99	8.38	93.45	-20.86	286.83	21.45
SSP370	2040s	160.64		172.08	69.95	18.48	102.90	-12.86	333.49	41.21
	2060s	145.50		146.43	67.20	13.81	100.53	-14.86	313.23	32.63
	2080s	122.10		106.80	62.70	6.19	93.13	-21.13	277.92	17.68
	2100s	114.42		93.79	66.31	12.31	99.25	-15.95	279.97	18.55
SSP585	2040s	146.06		147.39	69.01	16.89	100.98	-14.49	316.05	33.82
	2060s	150.42		154.76	67.62	14.53	97.28	-17.62	315.32	33.51
	2080s	135.47		129.45	61.93	4.89	89.72	-24.02	287.13	21.58
	2100s	131.30		122.38	61.78	4.64	87.05	-26.28	280.13	18.61

The most pronounced changes under future scenarios occur in highly suitable habitats. Compared with the present, the area of highly suitable habitats increases substantially across all scenarios, with the highest increase reaching 185%. Particularly during the early stages of SSP126 and SSP370 (2021–2040), peak growth was observed in highly suitable habitats. In contrast, marginally suitable habitat shows an overall decline, with reductions ranging from 10% to 26% across most scenarios. Moderately suitable habitat exhibits slight increases or fluctuating changes overall, with relatively stable growth rates.

From a temporal perspective, under the low-to-medium emissions scenario (SSP126), the expansion of suitable habitats remains relatively stable. Conversely, under high emissions scenarios (SSP370 and SSP585), although significant early expansion occurs, the rate of increase gradually diminishes after 2060, with some periods even showing a decline. This suggests that excessive warming may exceed the species’ physiological tolerance, thereby constraining its expansion.

**Figure 7:**
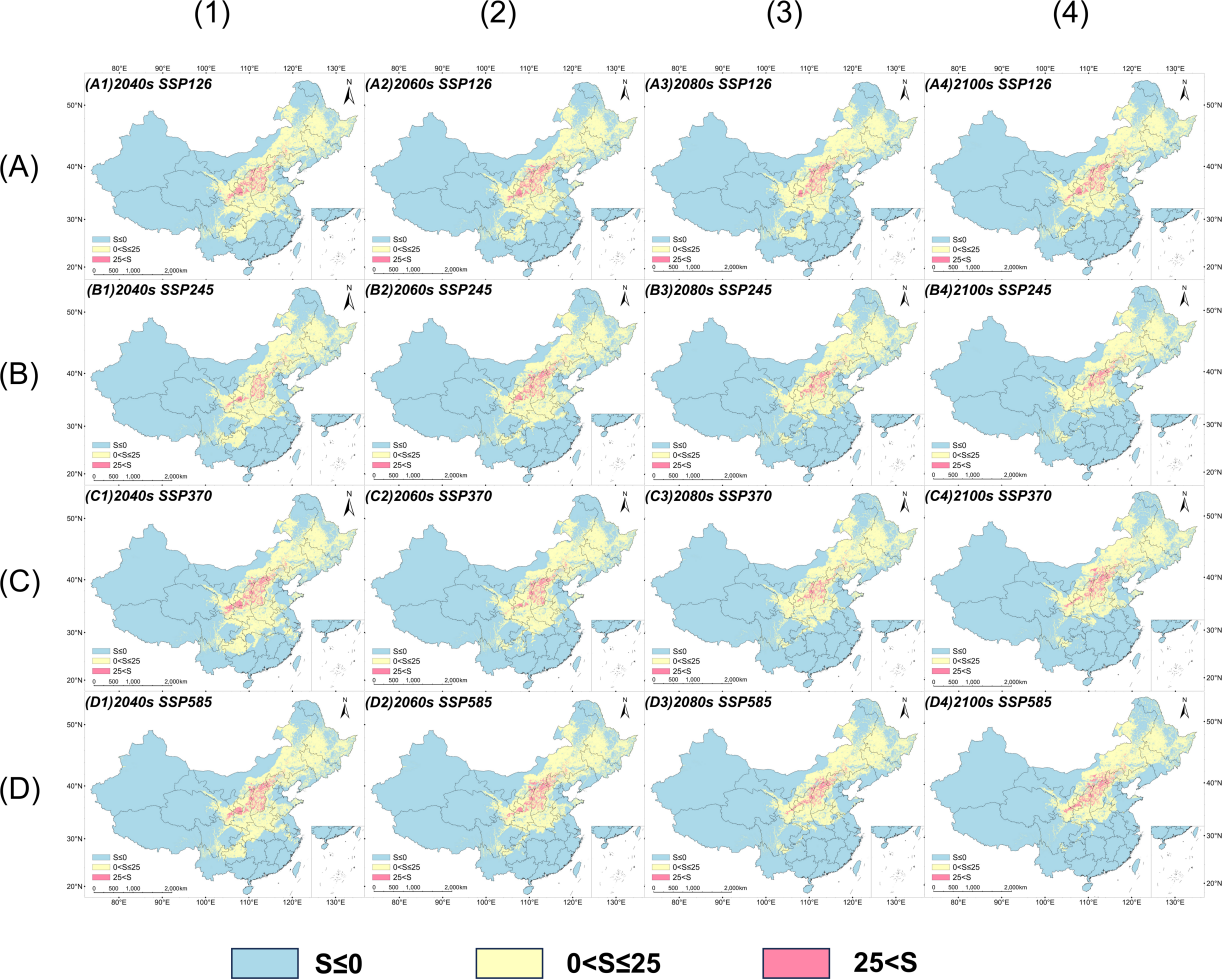
MESS analysis for *V. nigrum* under future climate scenarios: **(A)** SSP126, **(B)** SSP245, **(C)** SSP370, and **(D)** SSP585 climate scenarios; (1) 2040s, (2) 2060s, (3) 2080s, and (4) 2100s.

Furthermore, results from the Multivariate Environmental Similarity Surface (MESS) analysis (**Figure 7**) indicate that most future suitable areas fall within the training range of environmental variables (S > 0), suggesting that the model predictions possess high overall reliability. However, under various scenarios and time periods, regions in northern Inner Mongolia Autonomous Region and northern Heilongjiang Province exhibit S ≤ 0, suggesting that environmental conditions in these areas exceed the scope of current model training data. Consequently, predictions for these regions should be interpreted with caution.

Overall, future climate change may promote the expansion of *V. nigrum*’s potential distribution range, particularly with a significant increase in core high-suitability habitat. However, prolonged warming under high-emission scenarios may attenuate this expansion trend. This indicates the species exhibits strong responsiveness to climate change, with its future distribution patterns undergoing dynamic adjustments according to climate scenarios and temporal scales.

### 3.4 Centroid migration of suitable habitat

**Figure 8:**
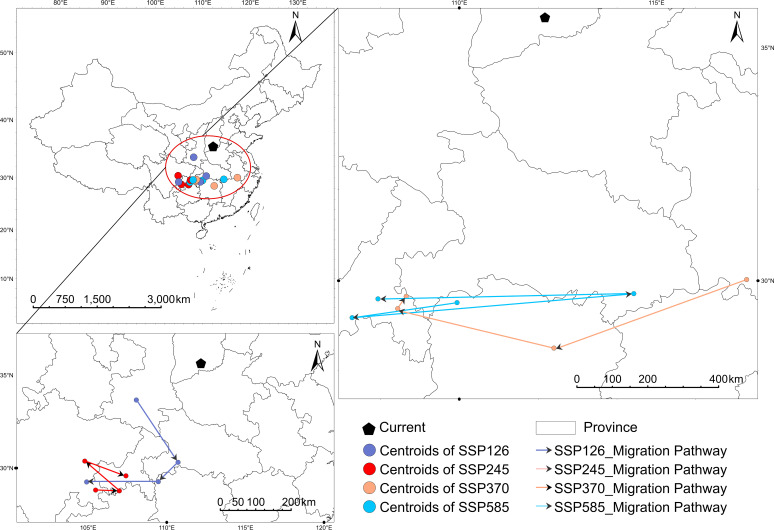
Centroid migration of *V. nigrum* under different climate scenarios.

To quantify the dynamic trends in the spatial centroid of *V. nigrum*’s suitable habitat under future climate change, ArcGIS Pro was employed to determine the centroid locations of suitable habitats for the present and under various SSP scenarios across different time periods (**Figure 8**).

Under the current scenario, the habitat suitability centroid is situated in southern Shanxi Province (approximately 112.17^°^E, 35.64^°^N), within the transitional zone between North China and the Loess Plateau. Under future scenarios, the habitat suitability centroid shows a pronounced overall spatial migration trend.

By the 2021–2040 period, the centroids in all scenarios had shifted southwards. Specifically: while under SSP126 and SSP585, the centroids were situated in the transitional zone from southern Shaanxi to northern Chongqing Province. Under SSP370, the centroid shifted slightly westward, positioning near the border between Shanxi and Shaanxi provinces. By 2041, the centroids in all scenarios had shifted further southwest or westward, concentrating predominantly in central-southern Shaanxi and western Shanxi.

By the 2061 phase, differences between emission scenarios became apparent: under SSP245, the centroid shifted slightly northeastwards, while under SSP585 it moved northwestwards into the transitional zone between southern Ningxia and eastern Gansu. This phase indicates divergence in migration trajectories under high-emission scenarios. By 2081, the centroids stabilise across scenarios, predominantly distributed across central Shaanxi and western Shanxi (approximately 104^°^E–108^°^E, 29^°^N–30^°^N). Compared with the current centroid, this period exhibits a pronounced southwesterly migration trend, with a net southwesterly displacement of approximately 5–6^°^ in latitude and minor longitudinal shifts (<3^°^).

Overall, future climate change may cause the centroid of *V. nigrum*’s suitable habitat to shift from North China towards the southwestern-western transition zone, with the dominant direction reflecting the combined effects of southward and westward movements. Centroid migration remains relatively stable under medium-to-low emission scenarios, whereas the high-emission scenarios exhibit greater pathway uncertainty, indicating greater uncertainty in future migration pathways.

### 3.5 Spatial characteristics and quantitative analysis of the distribution risk zones for *V. nigrum* and three livestock species

**Figure 9:**
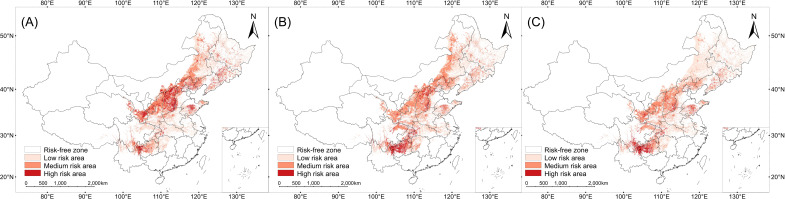
Spatial risk partitioning of *V. nigrum* distribution in livestock husbandry: **(A)** sheep, **(B)** goats, **(C)** cattle.

Through spatial overlay and quantitative analysis of the distribution density of *V. nigrum* with sheep, cattle, and goats, the results indicate (**Figure 9**) that the spatial patterns of risk zones for the three livestock species exhibit distinct differences. The cattle risk zone exhibits the most extensive coverage, spanning a total area of 83.54 × 10^4^ km². It displays a distribution pattern characterised by a core concentration in the southwestern region and localised to high-elevation microhabitats across northern agricultural zones, with its core risk zones concentrated in the Sichuan Basin and surrounding provinces, extending northwards along the Yangtze River basin into the Qinling Mountains, Hengduan Mountains, Tibetan Plateau margins. This reflects the extensive overlap between cattle farming areas and the suitable habitat range of *V. nigrum* across the entire region.

Conversely, the risk zones for sheep and goats are more geographically constrained. The sheep risk zone (80.98 × 10^4^ km²) closely follows China’s northern agro-pastoral transition zone and the southwestern pastoral fringes. In contrast, the goats risk zone exhibits the smallest spatial extent, covering only 68.04 × 10^4^ km², with a distinctly localised and fragmented distribution pattern. Its risk areas are primarily isolated within the Sichuan Basin and northern Yunnan-Guizhou Plateau, while its northern extension zone is markedly narrower and exhibits a fragmented morphology. In summary, distinct spatial patterns emerge across livestock types within risk zones, with their spatial evolution closely tied to the distribution of respective livestock farming practices.

## 4 Discussion

### 4.1 Environmental drivers of *V. nigrum* distribution

The spatial distribution of *V. nigrum* is typically influenced by multiple environmental variables, which alter growth conditions and resource availability, thereby determining its distribution pattern. The findings of this study indicate that wet season precipitation (bio13), mean temperature of the driest season (bio9), and altitude are the core variables shaping the suitability pattern for *V. nigrum*. Different environmental variables contain independent information and may also interact with one another. For instance, the combined variation of moisture and temperature conditions affects plant growth, thereby influencing its distribution pattern and suitable habitat range within temperate mountain ecosystems.

Bio13 (Precipitation of Wettest Month) reflects the region’s moisture supply capacity and abundance during the wet season. Research indicates that wet-season precipitation directly influences plant water balance (Wu et al., 2022). Adequate moisture enables plants to maintain higher water potential during the growing season, thereby promoting growth. Should abnormal drought occur during the mid-to-late wet season, water accumulated earlier in soil and plant tissues can partially buffer water deficits and mitigate drought impacts. This mechanism is commonly observed in perennial herbs. As a perennial herb, *V. nigrum* exhibits heightened dependence on wet-season precipitation. Precipitation variations influence the plant’s water relationship and stress tolerance by regulating key physiological indicators such as the point of turgor pressure loss (Ψ_tlp_), photosynthetic rate, and osmotic regulation (Grossman, 2022).

Bio9 (Mean Temperature of Driest Quarter) reflects thermal conditions during the dry season. Response curves indicate that *V. nigrum* exhibits a higher probability of occurrence at lower temperatures, with a significant decline as temperatures rise. High temperatures during the dry season intensify plant water loss, thereby inhibiting the growth of *V. nigrum*. In water-limited areas, plants expend greater metabolic energy to cope with water stress (Sadok et al., 2020). The synergistic effects of extreme heat and drought significantly suppress perennial herbaceous growth, a phenomenon particularly pronounced in temperate regions (Wang and Wang, 2023).

Altitudinal gradients in environmental variables such as climate, temperature, and precipitation directly influence plant distribution patterns. As elevation increases, temperatures decrease and precipitation patterns shift, driving systematic changes in species distribution. The genus *Veratrum* is predominantly distributed within the altitude range of 400–4000 metres (Gu et al., 2018). The low-temperature environment and short growing season at high altitudes significantly alter plant growth rhythms and shape unique adaptive strategies. In high-altitude environments, *V. nigrum* populations often reduce reproductive investment and enhance photosynthetic efficiency to regulate energy allocation. This strategy may shorten flowering intervals (Ito and Kudo, 2024). However, excessive elevation induces multiple stresses that inhibit perennial herb growth. For instance, *Salvia przewalskii* adapts to altitude changes by regulating leaf physiological and biochemical traits. High-altitude habitats constrain carbon assimilation in Salvia gansuensis leaves, limiting this process at elevated elevations. To sustain survival, plants adjust resource allocation by reducing organic synthesis and directing more resources towards stress defence systems, thereby balancing growth and survival (Xing et al., 2024).

### 4.2 Impacts of climate change on future distribution

Under future climate scenarios, *V. nigrum* is projected to expand its overall distribution, with increases in both total and highly suitable habitats across most SSP scenarios. This indicates a substantial reshaping of its distribution pattern. However, this response does not represent a simple linear expansion, as it is jointly influenced by climatic stress, physiological responses, and regional environmental heterogeneity.

In addition to changes in overall area, climate change drives notable spatial adjustments. The centroid of *V. nigrum*’s suitable habitat shifted 5–6^°^ latitude toward the southwest and west, reflecting a realignment of the species’ niche with changing thermohygric conditions (Li et al., 2019; Hu et al., 2023). This migration indicates that climate change affects not only the extent of suitable habitat but also its spatial configuration. The redistribution of habitat quality further highlights the importance of core areas. Higher water-use efficiency enables plants to maintain stability under intensified aridity, supporting the expansion of core suitable habitats in ecologically favourable regions (Zhen et al., 2024). This concentration may increase local population density and influence community interactions.

Meta-analyses show that the combined effects of warming and drought suppress carbon metabolism more strongly than individual stressors (Wang and Wang, 2023); meanwhile, as a C_3_ herbaceous species, *V. nigrum* may still benefit to some extent from elevated atmospheric CO_2_ through enhanced carbon assimilation and improved water-use efficiency (Liu et al., 2004; Mokhtar et al., 2025). Therefore, although climate change generally promotes expansion, the positive effects of warming may be moderated under high-emission scenarios as heat and water stress reduce the magnitude of projected habitat gains.

### 4.3 Implications for grazing management and livestock risk

Overlap between the suitable habitat of *V. nigrum* and the spatial distribution of grazing livestock may amplify the risks posed by toxic weeds to ruminant health and grassland ecosystems. Multiple systematic reviews in recent years have highlighted that toxic plants not only pose acute and chronic toxicity to ruminants via their secondary metabolites but also indirectly affect grazing animal health and productivity through grassland community structure and nutrient resource dynamics (Abdisa and Dilbato Dinbiso, 2024). Toxic compounds in poisonous plants—such as alkaloids, pyrrolizidine alkaloids, and other secondary metabolites—exhibit dynamic variations within plant tissues across growth stages, environmental conditions, and stress responses. These secondary metabolites, functioning as defensive chemical constituents, influence selective feeding behaviour in ruminants. In foraging systems characterised by feed scarcity or intense resource competition, they heighten exposure risks through increased consumption, thereby causing reduced productivity and impaired health (Rasool et al., 2024).

From a chemical ecology perspective, toxic plants typically modulate their secondary metabolic pathways following grazing or disturbance to enhance anti-grazing defences (e.g., by accumulating alkaloids, saponins, and other secondary compounds). Consequently, increased levels of these defensive chemicals under stress further inhibit animal consumption and suppress growth of other plants, stabilising toxic populations and perpetuating localised risks (Green et al., 2023).

Furthermore, evidence suggests that exposure risks to toxic plants may be modulated by variables such as grazing animal species and seasonal nutritional requirements. For instance, variations in tolerance to identical toxins and differing feeding selection behaviours among ruminants provide an ecologically behavioural layer to explain spatial pattern differences in risk zones for cattle, sheep, and goats.

### 4.4 Study limitations and future directions

Although the model developed in this study provides a relatively reliable projection of the suitable habitat for *V. nigrum*, several limitations remain. In particular, interspecific interactions were not considered, and incorporating species relationships in future work could improve the accuracy of distribution predictions. In addition, the analysis relied on a single climate model. While four emission scenarios were included, the use of multiple climate models would allow for a more comprehensive assessment of potential distributions. For risk assessment, future studies could further refine the spatial distribution of toxic component concentrations in *V. nigrum*, thereby improving the precision of risk evaluation.

## 5 Conclusion

Against a background of intensifying climate change functions, this study projects the potential distribution of *V. nigrum* and evaluates its risks to livestock farming. To improve model performance, NSGA-III was incorporated to optimise MaxEnt model selection across multiple objectives. This multi-objective framework helps balance model discriminative power, overfitting, and complexity, ultimately improving the reliability of the predictions. From an ecological perspective, the distribution of *V. nigrum* is primarily influenced by the combined effects of topography and hydrothermal conditions. Water availability during the wet season, thermal conditions during the dry season, and altitude collectively shape its ecological niche preference for medium-to-high elevations, relatively humid environments, and cooler dry seasons. Under future climate scenarios, the potential habitat of *V. nigrum* shows an overall expansion trend accompanied by a shift in its distribution centroid, with some marginally suitable areas gradually transitioning into moderately or highly suitable zones. Spatial overlay analysis between *V. nigrum*’s suitable areas and livestock distribution densities indicates varying risks across different livestock species. Risk zones for cattle, sheep, and goats exhibit distinct characteristics in both area and spatial morphology, suggesting that grassland risk prevention requires more targeted management strategies. For areas with high model extrapolation uncertainty, risk assessments should be conducted using a combination of conservative constraints and continuous monitoring. Overall, this study provides spatial evidence for resource management, grassland ecological early warning, and grazing management for plants with both medicinal value and toxic risk under global change.

## Data Availability

The original contributions presented in the study are included in the article/**Supplementary Material**. Further inquiries can be directed to the corresponding author.
