# Supplementary material for: Potential distribution, range dynamics, and livestock exposure risk of Veratrum nigrum L. in China under climate change
Source: Front Plant Sci. 2026 May 13;17:1835288. doi: 10.3389/fpls.2026.1835288 (PMC13212438; doi:10.3389/fpls.2026.1835288)
Supplement: Supplementary file 1 [file Table1.docx]

Supplementary Material

# Supplementary Figures and Tables

## Supplementary Figures


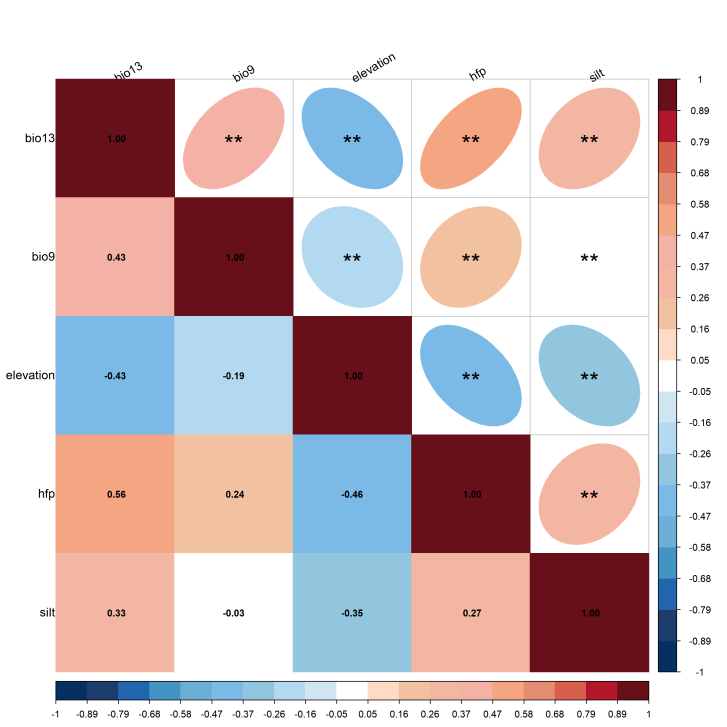


**Figure S1.** Correlation matrix of the 5 environmental variables. * denotes p < 0.05.** denotes p < 0.01.

## Supplementary Tables

**Table S1.** Bioclimatic, soil, topographic, human footprint, and livestock density variables utilised in this study

| **Variable code** | **Environmental Variables** |
| --- | --- |
| bio1 | Annual Mean Temperature (°C) |
| bio2 | Mean Diurnal Range (Mean of monthly (maximum temperature - minimum temperature)) (°C) |
| bio3 | Isothermality (BIO2/BIO7×100) |
| bio4 | Temperature Seasonality (standard deviation ×100) (°C) |
| bio5 | Maximum Temperature of Warmest Month (°C) |
| bio6 | Minimum Temperature of Coldest Month (°C) |
| bio7 | Annual Temperature Range (BIO5-BIO6) (°C) |
| bio8 | Mean Temperature of Wettest Quarter (°C) |
| bio9 | Mean Temperature of Driest Quarter (°C) |
| bio10 | Mean Temperature of Warmest Quarter (°C) |
| bio11 | Mean Temperature of Coldest Quarter (°C) |
| bio12 | Annual Precipitation (mm) |
| bio13 | Precipitation of Wettest Month (mm) |
| bio14 | Precipitation of Driest Month (mm) |
| bio15 | Precipitation Seasonality (Coefficient of Variation) |
| bio16 | Precipitation of Wettest Quarter (mm) |
| bio17 | Precipitation of Driest Quarter (mm) |
| bio18 | Precipitation of Warmest Quarter (mm) |
| bio19 | Precipitation of Coldest Quarter (mm) |
| clay | Clay |
| cn_ratio | Carbon-to-nitrogen ratio (C/N) |
| coarse | Coarse fragments |
| org_carbon | Organic carbon content |
| pH in water | pH in water |
| ref_bulk | Reference bulk density (g/cm³) |
| sand | Sand |
| silt | Silt |
| teb | Teb (cmolc/kg) |
| total_n | Total nitrogen content (g/kg) |
| elevation | Elevation (m) |
| aspect | Aspect (◦) |
|  |  |
| Slope | Slope (◦) |
| hfp | Human Footprint Index |
| goats | Annual goats density (heads/km²) |
| sheep | Annual sheep density (heads/km²) |
| cattle | Annual cattle density (heads/km²) |

**Table S2.** Variance Inflation Factor (VIF) values of the five environmental variables used for multicollinearity assessment.

| Variables | VIF |
| --- | --- |
| bio13 | 1.944100 |
| bio9 | 1.682434 |
| elevation | 1.391644 |
| hfp | 1.517732 |
| silt | 1.352792 |
